# Contact tracing for tuberculosis, Thailand

**DOI:** 10.2471/BLT.19.239293

**Published:** 2020-01-27

**Authors:** Worarat Imsanguan, Surasit Bupachat, Vanichaya Wanchaithanawong, Sarmwai Luangjina, Sureerat Thawtheong, Supalert Nedsuwan, Petchawan Pungrassami, Surakameth Mahasirimongkol, Amornrat Wiriyaprasobchok, Kulayanee Kaewmamuang, Phalin Kamolwat, Jintana Ngamvithayapong-Yanai

**Affiliations:** a Chiangrai Prachanukroh Hospital, Chiang Rai, Thailand.; b TB/HIV Research Foundation, 1050/1 Satarnpayabarn Rd., Muang District, Chiang Rai 57000, Thailand.; c Ministry of Public Health, Nonthaburi, Thailand.

## Abstract

**Problem:**

Despite implementation of universal health coverage in Thailand, gaps remain in the system for screening contacts of tuberculosis patients.

**Approach:**

We designed broader criteria for contact investigation and new screening practices and assessed the approach in a programme-based operational research study in 2017–2018. Clinic staff interviewed 100 index patients and asked them to give household and non-household contacts an invitation for a free screening and chest X-ray. Contact persons who attended received 250 Thai baht (about 8 United States dollars) allowance for transport.

**Local setting:**

Chiang Rai province, Thailand, has high rates of tuberculosis notification and a high number of people living in poverty. The coverage of contact investigation in under 5-year-olds was only 33.2% (222 screened out of 668 contacts) over 2011–2015.

**Relevant changes:**

Index patients identified 440 contacts in total and gave invitation cards to 227 of them. The contact investigation coverage was 81.1% (184/227) and tuberculosis detection among contacts screened was 6.0% (11/184). Of the 11 contacts with active tuberculosis, three did not have tuberculosis symptoms, three were non-household contacts and three were contacts of non-smear-positive tuberculosis patients. The contact investigation coverage of the contacts younger than 5 years was 100% (14/14) and the yield of tuberculosis detection in this age group was 21.4% (3/14).

**Lessons learnt:**

High coverage of contact investigation with a high yield of tuberculosis detection among contacts can be achieved by applying broader criteria for contact investigation and providing financial support for transportation.

## Résumé

**Problème:**

Malgré la mise en œuvre d'une couverture maladie universelle en Thaïlande, des lacunes subsistent dans le système de dépistage des contacts de patients atteints de la tuberculose.

**Approche:**

Nous avons défini des critères élargis pour l'enquête d'entourage et les nouvelles pratiques de dépistage, puis évalué cette approche par le biais d'un programme de recherches menées sur le terrain en 2017–2018. Le personnel médical a interrogé 100 patients de référence et leur a demandé de transmettre à leurs contacts issus de l'environnement familial et extra-familial une invitation à un dépistage et à une radiographie du thorax, tous deux gratuits. Les contacts qui se rendaient à ces examens recevaient 250 bahts thaïlandais (l'équivalent de 8 dollars américains environ) d'indemnité de transport.

**Environnement local:**

La province thaïlandaise de Chiang Rai affiche un fort taux de cas de tuberculose et un nombre élevé de personnes vivant dans la pauvreté. L'enquête d'entourage n'a touché que 33,2 % des moins de 5 ans (222 dépistages sur les 668 contacts) durant la période comprise entre 2011 et 2015.

**Changements significatifs:**

Les patients de référence ont identifié 440 contacts au total, et ont distribué 227 invitations. L'enquête d'entourage a porté sur 81,1 % de ces contacts (184/227) et la tuberculose a été diagnostiquée chez 6,0 % d'entre eux (11/184). Sur les 11 contacts atteints de la tuberculose, trois ne présentaient aucun symptôme, trois provenaient de l'environnement extra-familial et trois étaient en contact avec des patients souffrant de tuberculose mais n'étant pas des cas à frottis positif. L'enquête d'entourage a touché 100 % des contacts de moins de 5 ans (14/14) ; la tuberculose a été détectée chez 21,4 % des contacts appartenant à cette tranche d'âge (3/14).

**Leçons tirées:**

Il est possible de couvrir un grand nombre de contacts avec l'enquête d'entourage et de détecter un maximum de cas en appliquant des critères élargis et en fournissant une aide financière pour les transports.

## Resumen

**Situación:**

A pesar de la implementación de la cobertura sanitaria universal en Tailandia, siguen existiendo deficiencias en el sistema de detección de los contactos de pacientes con tuberculosis.

**Enfoque:**

Se diseñaron criterios más amplios para la investigación de los contactos y las prácticas de cribaje nuevas y se evaluó el enfoque en un estudio de investigación operativa basado en un programa de 2017 a 2018. El personal de la clínica entrevistó a 100 pacientes índice y les solicitó que invitaran a los contactos domésticos y no domésticos a un cribado y una radiografía de tórax gratuitos. Las personas de contacto que asistieron recibieron un subsidio de 250 baht tailandeses (unos 8 dólares estadounidenses) para el transporte.

**Marco regional:**

La provincia de Chiang Rai, Tailandia, tiene tasas altas de notificación de tuberculosis y un número elevado de personas que viven en la pobreza. La cobertura de la investigación de contactos en menores de 5 años fue de solo el 33,2 % (222 cribados de 668 contactos) en el periodo 2011-2015.

**Cambios importantes:**

Los pacientes índice identificaron 440 contactos en total y entregaron tarjetas de invitación a 227 de ellos. La cobertura de la investigación de contactos fue del 81,1 % (184/227) y la detección de la tuberculosis entre los contactos examinados fue del 6,0 % (11/184). De los 11 contactos con tuberculosis activa, tres no presentaban síntomas de tuberculosis, tres eran contactos no domésticos y tres eran contactos de pacientes con tuberculosis no baciloscópica. La cobertura de la investigación de los contactos menores de 5 años fue del 100 % (14/14) y el rendimiento de la detección de la tuberculosis en este grupo de edad fue del 21,4 % (3/14).

**Lecciones aprendidas:**

Se puede lograr una alta cobertura de la investigación de contactos con un alto rendimiento de la detección de la tuberculosis entre los contactos aplicando criterios más amplios para la investigación de contactos y ofreciendo apoyo financiero para el transporte.

## ملخص

**المشكلة:**

على الرغم من تنفيذ التغطية الصحية الشاملة في تايلند، ما زالت هناك فجوات في نظام فحص الأشخاص المتصلين بمرضى السل.

**الأسلوب:**

قمنا بتصميم معايير أوسع لفحص الأشخاص المتصلين، وممارسات الفحص الجديدة، قمنا بتقييم الأسلوب في دراسة بحثية تشغيلية قائمة على البرامج في الفترة 2017 إلى 2018. قام فريق عمل العيادة بمقابلة 100 من المرضى ذوي المؤشرات، وطلبوا منهم دعوة الأشخاص المتعاملين معهم سواء بالمنزل أو خارجه، لإجراء فحص مجاني، وتصوير الصدر بالأشعة السينية. حصل الأشخاص المتصلون الذين حضروا على 250 بهت تايلندي (حوالي 8 دولارات أمريكية) كبدل للنقل.

**المواقع المحلية:**

توجد ****في مقاطعة شيانج راي، في تايلند، معدلات مرتفعة من بلاغات مرض السل، وعدد كبير من الأشخاص الذين يعيشون في فقر. كانت تغطية فحص الأشخاص المتصلين الأقل من 5 سنوات، 33.2% فقط (تم فرز 222 من أصل 668 شخص متصل) خلال الفترة من 2011 إلى 2015.

**التغييرات ذات الصلة:**

حدد المرضى من ذوي المؤشرات إجمالي 440 شخصاً من المتصلين، وقدموا بطاقات دعوة إلى 227 منهم. كانت تغطية فحص الأشخاص المتصلين 81.1% (184/227)، وتم اكتشاف الإصابة بالسل بين الأشخاص المتصلين بنسبة 6.0% (11/184). من بين 11 شخصاً من المتصلين بمرضى السل النشط، لم تظهر لدى ثلاثة منهم أعراض مرض السل، وثلاثة منهم كانوا من خارج المنزل، وثلاثة آخرون تم تشخيص إصابتهم بالسل عن طريق مسحات البصاق الإيجابية. كانت تغطية فحص الأشخاص المتصلين للأشخاص الأصغر من 5 سنوات هي 100% (14/14)، وكانت حصيلة الكشف عن السل في هذه الفئة العمرية 21.4% (3/14).

**الدروس المستفادة:**

يمكن تحقيق تغطية عالية لفحص الأشخاص المتصلين بالمرضى، مع حصيلة عالية من الكشف عن السل بين هؤلاء الأشخاص، عن طريق تطبيق معايير أوسع لفحص الأشخاص المتصلين بالمرضى، وتقديم الدعم المالي للنقل.

## 摘要

**问题:**

尽管泰国实施了全民健康覆盖，但在筛查结核病患者接触者的系统中仍存在差距。

**方法:**

我们为接触调查和新的筛选实践设计了更广泛的标准，并于 2017 至 2018 年在一项运筹学研究项目中对该方法进行了评估。诊所工作人员采访了 100 名索引患者，并要求他们向家庭和非家庭接触者发出免费筛查和胸部 X 光检查的邀请。接受邀请的接触者会获得 250 泰铢（约 8 美元）的交通津贴。

**当地状况:**

泰国清莱府的结核病通报率很高，贫困人口也很多。在 2011 至 2015 年期间，5 岁以下儿童接触调查的覆盖率仅为 33.2%（668 名接触者中筛选出 222 人)。

**相关变化:**

索引患者总共确认了 440 名接触者，并向其中 227 人发出了邀请卡。接触调查覆盖率为 81.1% (184/227)，筛查的接触者结核检出率为 6.0% (11/184)。在 11 例活动性结核病患者中，3 例无结核症状，3 例为非家庭接触者，还有 3 例为非涂阳结核患者接触者。5 岁以下接触者的接触调查覆盖率为 100%（14/14），该年龄组结核病检出率为 21.4%（3/14）。

**经验教训:**

采用更广泛的接触调查标准，为交通运输提供资金支持，可以实现较高的接触调查覆盖率和接触者结核病的检出率。****

## Резюме

**Проблема:**

Несмотря на внедрение в Таиланде системы всеобщего охвата услугами здравоохранения, в системе остаются недочеты применительно к выявлению лиц, контактирующих с больными туберкулезом.

**Подход:**

Авторы разработали расширенные критерии изучения лиц, контактирующих с больными, и новые практические принципы скрининга, а также провели оценку подхода в программном практическом исследовании в течение 2017–2018 гг. Персонал клиники опросил 100 нулевых пациентов и попросил их пригласить членов семьи и друзей посетить клинику для прохождения бесплатного скрининга и рентгена грудной клетки. Посетившим клинику лицам, находящимся в контакте с больными, выдавали по 250 тайских бат (около 8 долларов США) для покрытия транспортных расходов.

**Местные условия:**

В провинции Чианг Рай Таиланда отмечается высокий уровень заболеваемости туберкулезом в сочетании с высоким показателем нищеты. На протяжении 2011–2015 гг. охват обследованием лиц младше 5 лет составлял всего 33,2% (222 выявленных случая заболевания среди 668 лиц, находившихся в контакте с больными).

**Осуществленные перемены:**

Нулевые пациенты идентифицировали в общей сложности 440 контактировавших с больными лиц и пригласили посетить клинику 227 из них. Охват обследованием составил 81,1% (184 из 227 человек), туберкулез был выявлен у 6,0% (11 из 184 человек). Из 11 контактировавших с больными лиц с активной формой туберкулеза у троих не имелось симптомов туберкулеза, еще трое не являлись членами семей пациентов, а трое контактировали с больными туберкулезом без положительного результата микроскопии мазка мокроты. Для лиц младше 5 лет охват обследованием составил 100% (14 из 14 детей), и у 21,4% (3 из 14) обследованных в этой возрастной группе был выявлен туберкулез.

**Выводы:**

Высокого уровня охвата обследованиями контактирующих с больными лиц и высокого уровня выявления больных туберкулезом среди этих лиц можно достичь за счет расширения критериев обследования и обеспечения финансовой помощи для проезда в клинику.****

## Introduction

Tuberculosis is curable and preventable, typically at low cost, but every year approximately 10 million people worldwide fall sick with tuberculosis and more than 1 million die.^1^ In Thailand, an estimated 12 000 people die due to this preventable disease.

The World Health Organization (WHO) and the World Bank are using tuberculosis service coverage for monitoring global progress towards universal health coverage (UHC).^2^ To end the tuberculosis epidemic in a generation, the high tuberculosis burden countries should give priority investments to populations at highest risk of tuberculosis, including households and close contacts. Economic evaluations have demonstrated the cost–effectiveness of contact investigation for the prevention of tuberculosis as well as its economic benefits to society as a whole.^3^^–^^5^ Contact persons should have access to quality and rapid tuberculosis diagnosis, treatment and prevention services.^6^ Hence, contact investigation is an imperative intervention to facilitate the global End TB Strategy. 

At our referral hospital in Thailand we found a low coverage of contact screening in small children, the group at highest risk of contracting tuberculosis infection and developing active tuberculosis disease. In response, we carried out a programme-based operational research study aiming to increase the contact investigation coverage and yield of tuberculosis detection in contact persons and to obtain evidence for guiding contact investigation practices.

## Setting

Thailand is one of 14 countries with the highest burdens of tuberculosis, human immunodeficiency virus (HIV) and tuberculosis co-infection and multidrug-resistant tuberculosis.^1^ Chiang Rai, about 800 km from Bangkok, is the northernmost province of Thailand, with a provincial population of 1 287 615 and approximately 13% hill-tribe minorities. The province has been ranked as one of the poorest in Thailand. The Chiang Rai tuberculosis registry reported higher tuberculosis notifications (143 per 100 000 population), mortality (11.4%; 180 deaths among 1576 registered patients) and HIV co-infection (15.5%; 227 of 1466 patients) compared with national data in 2018. We conducted the study at Chiang Rai hospital, a referral hospital for 16 district hospitals in the province that also serves as a primary care unit and a district hospital for people living in the central district (244 311 population in 2018). Tuberculosis was detected in 4.8% (58 of 1200) contacts of smear-positive patients in Chiang Rai in 2003.^7^ In our setting, only 33.2% (222 of 668) contacts younger than 5 years were screened over 2011–2015. Diagnosis and treatment of tuberculosis is free of charge for Thai people under the UHC scheme. However, diagnosis by chest X-ray for the contacts of people with tuberculosis is free only for contacts with tuberculosis symptoms.

## Approach

Our team comprised government tuberculosis service providers, including clinicians and nurses, and a multidisciplinary research team from a nongovernment organization based in Chiang Rai. Based on our literature review, we designed an intervention that included new criteria and practices of contact investigation than previously used in Thailand (Table 1). For example, we broadened the criteria for contact investigation to include not only smear-positive patients and household contacts, but also other categories of tuberculosis and non-household contacts, according to WHO’s recommendations for contact investigation.^8^

**Table 1:** Comparison of previous and current criteria and practices of contact investigation and the justifications for change in Chiang Rai province, Thailand

Process	Previous practice	Practice in this study	Justification for change
Recruiting tuberculosis index patients	Emphasized smear-positive tuberculosis patients	Included smear-positive tuberculosis patients, multidrug-resistant tuberculosis patients, all types of tuberculosis in children younger than 5 years, and all types of tuberculosis in people with human immunodeficiency virus infection	WHO’s recommendations for contact investigation^8^
Recruiting tuberculosis contact persons	Emphasized household contacts. The staff simply instructed tuberculosis patients to bring everyone in their house to visit the tuberculosis clinic for screening	Included household contacts and non-household contacts. The staff carefully interviewed tuberculosis patients to obtain a list of contacts (age, sex and relation with tuberculosis patient). A contact was defined as a person who always stayed closely and spent time with a tuberculosis patient 4–8 hours a day during the previous 3 months, including contacts living in the same house or living outside the house, but regularly visiting the patient at home and other social gatherings (e.g. work, school)	Studies have shown that the transmission of tuberculosis to contacts occurred outside the house, such as at schools and workplaces^9^^,^^10^
Method of screening for active tuberculosis disease	Tuberculosis symptom screening. Contacts with symptoms were eligible for tuberculosis screening by chest radiography. Diagnosis for latent tuberculosis infection. Contacts younger than 18 years were given a tuberculin skin test	Interview for tuberculosis symptoms. Regardless of the tuberculosis symptoms, all contacts are eligible for tuberculosis screening by chest radiography	Tuberculosis prevalence surveys in Thailand and South East Asia showed that 50% of smear-positive tuberculosis patients and 65% of culture-confirmed tuberculosis patients did not report having tuberculosis symptoms^11^
Applying social interventions	Health-care staff verbally instructed the tuberculosis patients to bring every household member for a screening for tuberculosis	Health-care staff offered an invitation card to the tuberculosis patient to give to his or her contacts. There was one card for each contact. Each card had a unique identifier, which allowed staff to follow-up with the index patients when their contacts did not attend for screening. The invitation cards included non-stigmatized information about the need for tuberculosis screening, the curability and preventability of tuberculosis, the free service with a 250 Thai baht travel allowance (about US$ 8) and clinic service hours (Monday to Friday from 09.00 to 15.00 hours). The contact persons presented the cards when they came for screening. The staff called or visited the index patients’ home to ask why contacts did not visit the hospital. Staff could only investigate non-attendance for patients who gave consent for home visits to their houses and their contacts	A study in South Africa showed that invitation cards increased tuberculosis screening among household and non-household contacts, with a high yield of tuberculosis detection.^12^ In our setting, tuberculosis patients are poor and cannot afford transport costs to the hospital. Financial assistance for attendance improved tuberculosis treatment outcomes^13^

US$: United States dollars; WHO: World Health Organization.

From March 2017 to February 2018, we recruited 100 patients newly diagnosed with tuberculosis to the study (64 patients with smear-positive tuberculosis, 34 patients with HIV and tuberculosis co-infection, one patient with multidrug-resistant tuberculosis and one child younger than 5 years with tuberculosis). Trained staff members interviewed the index patients about their contacts and displayed a video explaining the importance of contact investigation. The staff offered invitation cards for each index case to invite up to six contacts (four household and two non-household contacts) for a free chest X-ray (usual cost to patients 200 Thai baht, THB; about 6 United States dollars, US$) and 250 THB allowance for transportation. Invitation cards were numbered so that we could link contacts to the index patient. The cards included information about the hours and location of the hospital, and non-stigmatized information about the need for tuberculosis screening and the curability, and preventability of tuberculosis. The invitation cards facilitated communication with the staff when the contact people reached the hospital and were also used as evidence to obtain the transportation fee. All of the contacts brought the cards with them to the hospital.

For each index patient we recorded the number of contacts identified, the number of invitation cards that he or she was willing to accept, the contacts who came to the clinic for screening and the outcomes of contact screening. We calculated the contact investigation coverage (percentage of contact persons receiving an invitation card who were screened for tuberculosis) and yield of tuberculosis detection (percentage of screened contacts who were diagnosed with active tuberculosis). Index patients were asked to provide a telephone number or address for contact persons so staff could call or visit to ask about the reasons for not going for screening.

The tuberculosis service staff was already experienced with conducting interviews about tuberculosis symptoms. We gave the staff an additional 2-day training on how to interview the patients sensitively and obtain information about household and non-household contacts who met the criteria. The staff referred all contact persons for a chest X-ray, regardless of the presence of symptoms. The radiologists interpreted the X-ray results without extra incentives. 

## Relevant changes

**Fig. 1:**
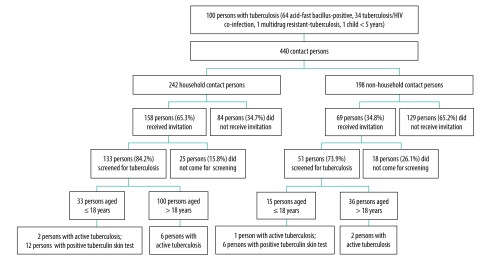
Contact investigation in children and adult contacts presenting results of screening by tuberculin skin test and chest X-ray in Chiang Rai province, Thailand

The index patients identified a total of 440 contact persons and gave invitation cards to 227 of them (Fig. 1). A total of 184 invited contacts came for screening, providing contact investigation coverage of 81.1%. Eleven of the contacts screened (6.0%) had active tuberculosis. Of these, 3 persons did not have tuberculosis symptoms, 3 persons were non-household contacts and 3 persons were contacts of non-smear-positive tuberculosis patients. Among the contacts younger than 5 years of age we achieved 100% coverage (14/14 contacts), followed by 90.2% (37/41 contacts) among contacts older than 60 years of age (Table 2). The yield of active tuberculosis in children younger than 5 years of age was also high (21.4%; 3/14 contacts), suggesting the need to ensure 100% coverage for small children contacts. The coverage of the tuberculin skin test for contacts under 18 years of age was 91.7% (44/48 contacts). 

HIV: human immunodeficiency virus.

Notes: Active tuberculosis indicates contact persons with or without tuberculosis symptoms diagnosed as tuberculosis based on result of a chest X-ray read by a radiologist and confirmed by a paediatrician or a pulmonologist.

**Table 2:** Coverage of contact investigation and yield of tuberculosis detection in contacts classified by age in Chiang Rai province, Thailand, 2017–2018

Age of contacts, years	No. of contacts receiving invitation card	No. (%) of contacts screened for tuberculosis	No. (%) of screened contacts diagnosed with active tuberculosis	Tuberculosis patients exposed to contacts
< 5	14	14 (100.0)	3 (21.4)	3 smear-positive patients
5–18	43	34 (79.1)	0 (0.0)	No contact found with active tuberculosis
19–60	126	99 (78.6)	3 (3.0)	1 smear-positive patient, 1 patient co-infected with tuberculosis and HIV,1 child with tuberculosis (4 years)
> 60	41	37 (90.2)	5 (13.5)	4 smear-positive patients, 1 patient co-infected with tuberculosis and HIV
Unknown	3	0 (0.0)	0 (0.0)	Unknown
**Total**	**227**	**184 (81.1)**	**11 (16.0)**	**8 smear-positive patients, 2 patients co-infected with tuberculosis and HIV, 1 child with tuberculosis (4 years)**

HIV: human immunodeficiency virus.

The reasons index patients gave for not accepting the contact invitation cards varied, including the contact having already been screened for tuberculosis or being on tuberculosis treatment, fearing losing their job, wanting to retain confidentiality about the disease, believing that they were not infected, and having conflicts with their contacts. Some patients could not locate the non-household contacts because they only temporarily attended the same training and did not know each other personally. The reasons contacts gave for not receiving a tuberculosis screening despite receiving an invitation card were: being busy with work, unwillingness to be screened for tuberculosis, inability to travel due to age or disability, not having anyone to accompany them, perceiving no risk for tuberculosis, mental health problems, and having moved to another place.

Based on the chest X-ray cost of 200 THB and transportation allowance of 250 THB, we calculated that the cost of detecting one contact with active tuberculosis was 7527 THB (184 contacts × 450 THB ÷ 11 cases of active tuberculosis). The cost equates to approximately US$ 254 if a transport allowance were provided to every contact, or US$ 119 if no travel expenses were provided. 

In November 2018, we had an opportunity to present our study findings to the Minister of Public Health of Thailand. In addition, our team members had several discussions with the public health authorities. As a result, the National Health Security Office, which is the organization responsible for implementing UHC in Thailand, agreed to cover the cost for tuberculosis screening as of October 2019 using chest X-ray for the household tuberculosis contact cases, regardless of the presence of tuberculosis symptoms. However, the other criteria and social interventions, which we applied in our study and increased tuberculosis case detection, have not yet been adopted by national health policy.

## Lessons learnt

Applying new criteria for recruiting tuberculosis patients, tuberculosis contacts and tuberculosis screening contributed to higher coverage, and a higher yield of detecting latent tuberculosis infection and active tuberculosis among the contacts of tuberculosis patients (Box 1). Despite the incentives offered, some contacts could not access tuberculosis screening due to challenges, such as poverty, stigma and health-system responses.^14^ If Thailand is to end the tuberculosis epidemic by 2035, the government and society must invest in contact investigation by addressing those challenges. The health system should improve health workers’ communication and interview skills as these skills will improve the coverage of contacts and the yield of tuberculosis detection.^14^^,^^15^ Some tuberculosis patients migrate for work to the capital city, Bangkok, but move to their hometown when they are sick. In this case, their contacts are in Bangkok and contact investigation should be performed by the local health service. Tuberculosis public health networks must be established and coordinated for contact investigation.

Box 1 **Summary of the main lessons learnt**

- Applying new criteria for recruiting tuberculosis patients and contacts and for tuberculosis screening contributed to higher coverage and a higher yield of detecting latent infection and active tuberculosis among patients’ contacts.
- Providing contacts with invitation cards and transport costs enhanced their access to tuberculosis screening, prevention and treatment.
- Tuberculosis contacts living with physical and mental disability and in extreme poverty require additional interventions to assist them in accessing tuberculosis screening and care.

Several studies, including our previous study,^14^ have reported that stigma against tuberculosis threatens contact investigation and the effort to end tuberculosis because index patients keep their illness confidential and conceal information about their contacts. As a result, contacts at high risk of tuberculosis, such as children younger than 5 years of age do not benefit from tuberculosis prevention and care. Campaigns to reduce tuberculosis stigma in the general population are urgently needed. We are now implementing a programme to reduce stigma against tuberculosis by means of education and communication strategies. To sustain the high contact investigation coverage and high yield of tuberculosis case detection achieved in this study, we are now engaging with multisectoral partners and civil society to support travel expenses for socioeconomically disadvantaged patients and their contacts.
