# Corrigendum

**DOI:** 10.2471/BLT.20.100420

**Published:** 2020-04-01

In: Imsanguan W., Bupachat S., Wanchaithanawong V., Luangjina S., Thawtheong S., Nedsuwan S., et al. Contact tracing for tuberculosis, Thailand. Bull World Health Organ. 2020 Mar 1;98(3):212–18 

On page 215, Table 2, should read as follows:

**Table 2:** Coverage of contact investigation and yield of tuberculosis detection in contacts classified by age in Chiang Rai province, Thailand, 2017–2018

Age of contacts, years	No. of contacts receiving invitation card	No. (%) of contacts screened for tuberculosis	No. (%) of screened contacts diagnosed with active tuberculosis	Tuberculosis patients exposed to contacts
< 5	14	14 (100.0)	3 (21.4)	3 smear-positive patients
5–18	43	34 (79.1)	0 (0.0)	No contact found with active tuberculosis
19–60	126	99 (78.6)	3 (3.0)	1 smear-positive patient, 1 patient co-infected with tuberculosis and HIV,1 child with tuberculosis (4 years)
> 60	41	37 (90.2)	5 (13.5)	4 smear-positive patients, 1 patient co-infected with tuberculosis and HIV
Unknown	3	0 (0.0)	0 (0.0)	Unknown
**Total**	**227**	**184 (81.1)**	**11 (6.0)**	**8 smear-positive patients, 2 patients co-infected with tuberculosis and HIV, 1 child with tuberculosis (4 years)**

HIV: human immunodeficiency virus.

